# The association between rheumatoid arthritis and reduced estimated cardiorespiratory fitness is mediated by physical symptoms and negative emotions: a cross-sectional study

**DOI:** 10.1007/s10067-023-06584-x

**Published:** 2023-03-24

**Authors:** Ingrid Sæther Houge, Mari Hoff, Vibeke Videm

**Affiliations:** 1 Department of Clinical and Molecular Medicine, NTNU – Norwegian University of Science and Technology, Trondheim, Norway; 2 Department of Neuromedicine and Movement Science, NTNU – Norwegian University of Science and Technology, Trondheim, Norway; 3 Department of Rheumatology, St. Olavs University Hospital, Trondheim, Norway; 4 Department of Immunology and Transfusion Medicine, St. Olavs University Hospital, Trondheim, Norway

**Keywords:** Cardiorespiratory fitness, Depressive symptoms, Mental health, Psychological stress, Rheumatoid arthritis

## Abstract

**Objectives:**

Persons with rheumatoid arthritis (RA) have lower cardiorespiratory fitness (CRF) than healthy individuals. We sought to identify variables explaining the association between RA status and reduced CRF.

**Methods:**

RA patients recruited from two Norwegian hospitals and blood donors recruited as controls filled in questionnaires about physical activity, physical symptoms, and psychological factors. Estimated CRF (eCRF) was calculated from non-exercise models. The relationship between RA status and reduced eCRF was explored with structural equation modelling. The latent variables physical symptoms (based on morning stiffness, joint pain, and pain in neck, back, or hips) and negative emotions (based on Hospital Anxiety and Depression Scale’s Depression score and Cohen’s perceived stress scale) were included as possible mediators between RA status and eCRF in separate and combined models adjusted for age and sex.

**Results:**

Two-hundred-and-twenty-seven RA patients and 300 controls participated. The patients were older and had lower eCRF than controls (age- and sex-adjusted mean difference: 1.7 mL/kg/min, p=0.002). Both latent variables were significant mediators of the association between RA and reduced eCRF when included in separate models. The latent variables mediated 74% of the total effect of RA on eCRF in the combined model. Standardized coefficients: direct effect of RA -0.024 (p=0.46), indirect effect through physical symptoms -0.034 (p=0.051), and indirect effect through negative emotions -0.034 (p=0.039).

**Conclusion:**

Both physical symptoms and negative emotions mediated the association between RA and reduced eCRF with similar effect sizes. To successfully increase CRF in RA patients, both physical and psychological factors should be addressed.

**Table:** 

**Key Points** *• The RA patients in the present study had 1.7 mL/kg/min lower mean estimated cardiorespiratory fitness (CRF) compared to healthy controls.* *• Mediation analysis demonstrated that physical symptoms and negative emotions mediated 74% of the total negative effect of RA on estimated CRF in a combined, adjusted model.* *• This suggests that both physical and psychological factors should be addressed when supporting RA patients in improving their CRF.*

**Supplementary Information:**

The online version contains supplementary material available at 10.1007/s10067-023-06584-x.

## Introduction

Cardiorespiratory fitness (CRF) is an important indicator of health and can be measured with a cardiopulmonary exercise test, or estimated using sub-maximal exercise tests or non-exercise models [1]. Low CRF is associated with increased risk of cardiovascular disease (CVD), depression, and diabetes, and with higher mortality rates [1, 2]. Rheumatoid arthritis (RA) is characterized by painful and swollen joints, morning stiffness, fatigue, and inflammation [3]. RA is also associated with increased risk of CVD and depression, and despite several reports of improved survival and some indicating similar survival as in the general population, RA was still associated with excess mortality in a recent nationwide Norwegian study [4–6]. RA patients with low CRF have more CVD risk factors and higher mortality rates than patients who are more physically fit [7, 8]. As RA patients have lower mean CRF compared to the general population, improving their fitness levels is an important part of managing their CVD risk profile [7, 9, 10].

Determinants of and correlates with CRF include sex, age, body composition, resting heart rate, physical activity (PA) habits, education, smoking habits, alcohol consumption, and genetics [11, 12]. Although the response to exercise may be influenced by individual factors, performing aerobic exercise is essential to improve and maintain CRF [1, 13]. Interventions involving regular aerobic exercise are associated with improved aerobic capacity, improved functional ability, reduced pain, and improved CVD risk profile in persons with RA [14, 15].

The European Alliance of Associations for Rheumatology (EULAR) emphasizes that PA and exercise should be an integrated part of standard care for persons with RA [16]. Consistent with the aerobic PA recommendations for the general population, EULAR recommends performing ≥150 minutes of PA at moderate intensity, or ≥75 minutes at vigorous intensity, or a combination of these, per week [16]. Psychological, physical, social, and environmental factors may affect PA behaviour and in turn CRF. RA patients have more joint symptoms and increased prevalence of depression compared to healthy individuals [4, 17]. Furthermore, RA patients tend to experience more work stress and interpersonal stressors than the general population, and the presence of RA can also be considered a stressor [18]. These factors may explain some aspects of the lower CRF estimates among RA patients compared to controls. For example, as psychological stress has been associated with a reduction in PA and exercise in longitudinal settings, it is plausible that higher levels of stress over time also lead to a reduction in fitness [19].

More knowledge on why RA patients have lower CRF levels than healthy individuals may identify areas that can be targeted in interventions or addressed in clinical practice. The hypothesis for the present study was that more physical symptoms and more negative emotions in RA patients would explain some of the association between RA status and lower estimated CRF (eCRF). The aim was to evaluate whether physical symptoms and/or negative emotions mediated the relationship between RA status and eCRF and to compare the relative effect of these two factors.

## Participants and methods

### Participants

The present study is part of a larger study, FysKond2, investigating the relationship between different patient-reported outcome measures and PA in patients with inflammatory joint diseases. Results from a subgroup of the participants who performed an optional 6-minute walk test have previously been published [20]. The present study included an RA group and a control group. To have large enough sample size to perform structural equation modelling (SEM), we aimed for at least 200 participants in each group.

**Fig. 1:**
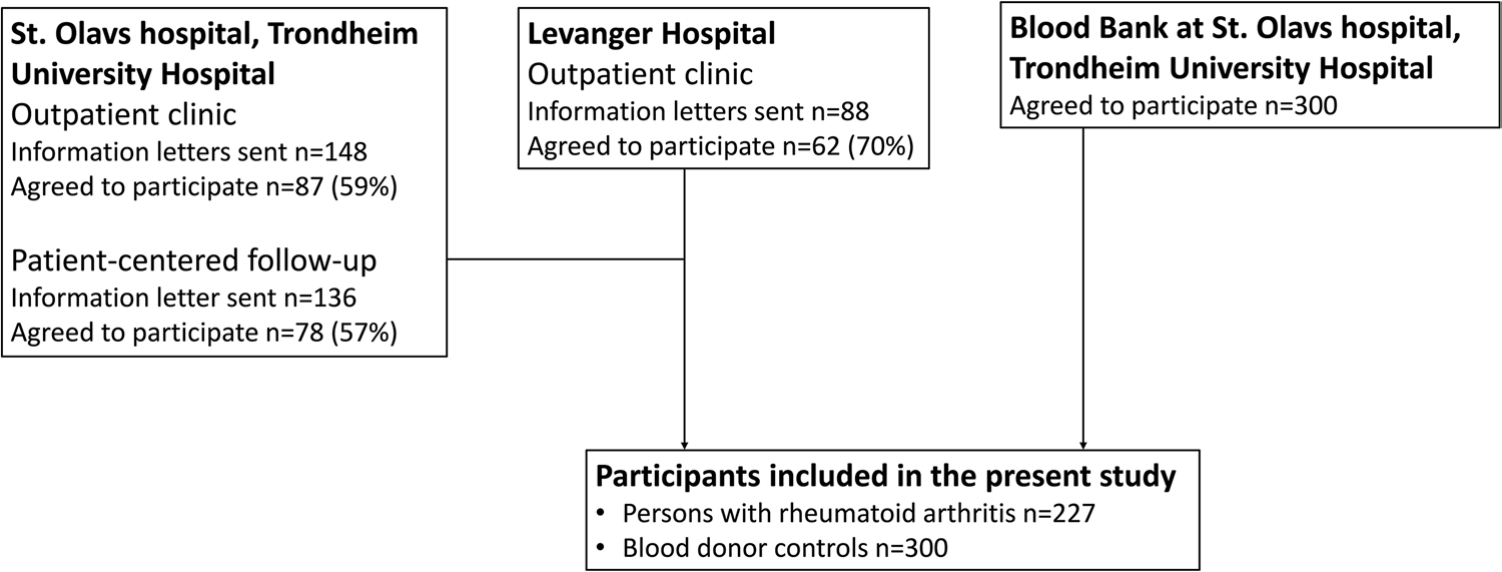
Inclusions to the study

Persons with an RA diagnosis according to the American College of Rheumatology/EULAR 2010 criteria were recruited at the Rheumatology Outpatient Clinics at Levanger Hospital and St. Olavs hospital, Trondheim University Hospital, Norway, in 2019 and 2021 [3]. All patients received an information letter about the study before they were contacted. At St. Olavs hospital, patients with well-controlled disease may be transferred to a patient-centred follow-up programme with regular controls at their general practitioner. To recruit a representative sample of RA patients, two recruitment strategies were used. We contacted a random selection of those in the patient-centred follow-up programme as well as patients with scheduled physical or digital appointments at the Rheumatology Outpatient Clinics during the recruitment periods, see Fig. 1. Persons with physical appointments were recruited during their visits. Those without a physical appointment were contacted by telephone and the questionnaires and a return envelope were sent to the participants by mail.

Blood donors were chosen as the control group because they are relatively easy to recruit and consist of persons of different ages and backgrounds. They fulfilled the national criteria for blood donors and were recruited in 2019 during an appointment at the Blood Bank at St. Olavs hospital [21].

### Questionnaires and data sources

All participants filled in questionnaires regarding PA, psychological factors, and physical symptoms, as further described below. Questions about frequency, duration, and intensity of performed PA were used to evaluate fulfilment of the aerobic PA recommendations and to calculate a PA index [16, 22]. Data regarding the rheumatic disease, use of anti-rheumatic medication, and comorbidities were collected from hospital records for the participants with RA. Due to the recruitment strategy explained above, many patients had not recently seen a rheumatologist and did not have a recent disease activity score of 28 joints (DAS28).

Non-exercise models developed in the same region of Norway were applied to calculate eCRF. An RA-specific model was used for the participants with RA and a model developed in healthy individuals was used for the controls [22, 23]. Both models include data on sex, age, body mass index, resting heart rate, and the PA index, and the RA model further includes smoking status and the patient global assessment [23, 24].

Degree of morning stiffness, intensity of joint pain in the past 6 months, and degree of pain in the neck, back, or hips were rated on a Likert scale from 0 to 10. A higher score indicates more severe symptoms.

The 10-item version of Cohen’s perceived stress scale was applied to assess level of stress [25]. The items include questions about the ability to handle stressors and emotional response to stressors in the past month. The scale ranges from 0 to 40, with higher scores indicating more perceived stress. The Hospital Anxiety and Depression Scale’s Depression score (HADS-D) was applied to assess the level of depressive symptoms [26]. The items include questions about mood, enjoyment etc. in the past week. HADS-D is based on 7 items and has a possible range of 0-21, with higher scores implying more depressive symptoms.

The participants with RA responded to the patient global assessment on a 100 mm visual analogue scale with the phrasing “Please consider the activity of your rheumatic disease in the past week. When considering all the symptoms, how do you think your state is?” Self-reported function in the past week was assessed with the modified Stanford Health Assessment Questionnaire (mHAQ) in the RA patients [27]. The mHAQ score has a possible range from 0.00 to 3.00, with higher scores indicating worse physical function. The RA patients also rated the degree of joint tenderness or swelling on a Likert scale from 0-10, with a higher score indicating more symptoms.

For participants who were recruited during a physical visit, resting heart rate was measured after the participant had been sitting for at least 5 minutes. Participants who mailed in their questionnaire were encouraged to count their resting heart rate after sitting for 10 minutes.

### Statistical analysis

Statistical analyses were performed using Stata (v16, StataCorp, College Station, TX, USA). P-values <0.05 were considered statistically significant. Normality was assessed with histograms and the Shapiro-Wilk test. Continuous data are presented as median with interquartile range or as mean with standard deviation as appropriate. Categorical data are presented as number with percentage. Comparisons of descriptive data were performed with the Mann-Whitney U test for non-normally distributed continuous data, the t-test for normally distributed continuous data, and the chi-square test for categorical data.

SEM was applied to evaluate the relationship between RA status and eCRF, including different potential mediators. SEM is a statistical method that tests whether a hypothesized model fits the observed data, and is suitable for mediation analysis, complex models, and models with latent variables. Mediation analysis tests whether some of the effect of the independent variable on the dependent variable is mediated by an intermediary variable (a mediator), meaning that the independent variable influences the mediator which in turn influences the dependent variable. A latent variable is not measured directly, but rather derived from numerous observed variables representing a common concept. SEM can include participants with missing data in the analysis, utilizing the available data. Furthermore, several dependent variables may be included in the same SEM model, and variables may correlate. In SEM several fit indices are used when evaluating whether the observed data fit with the proposed model. A model with good fit should have a non-significant chi-square test, root mean square error of approximation (RMSEA) <0.10, comparative fit index (CFI) ≥0.90, and Tucker-Lewis Index (TLI) ≥0.90 [28]. Several hypothesized models might fit the data adequately. Further details regarding SEM are provided in Online Resource Text 1. Both the unstandardized and standardized path coefficients were calculated. Following standardization, all coefficients in the model are given on the same scale, namely standard deviations for each included variable.

**Fig. 2:**
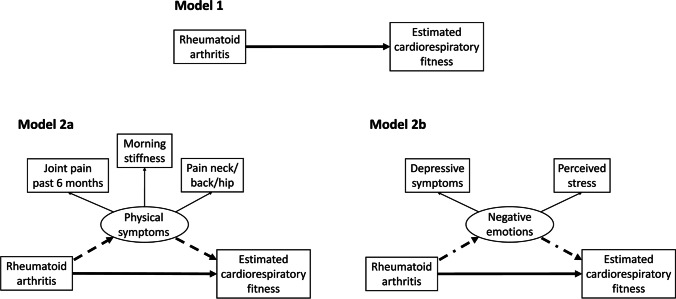
Pathway diagrams for Model 1, 2a and 2b. Structural equation models investigating the direct effect of the presence of rheumatoid arthritis on estimated cardiorespiratory fitness (──), and the indirect effect through either physical symptoms (----) or negative emotions (─ · ─). Latent variables are represented as circles, observed variables as squares, and pathways with arrows

**Fig. 3:**
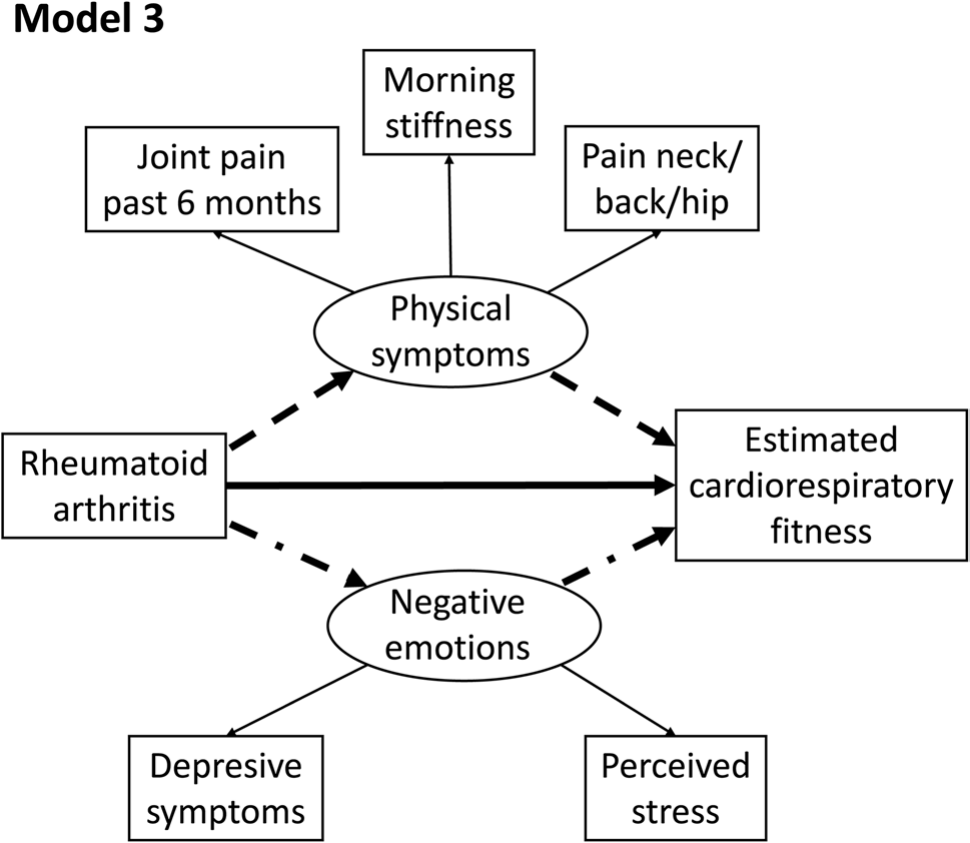
Pathway diagram for Model 3. Structural equation model investigating the direct effect of the presence rheumatoid arthritis on estimated cardiorespiratory fitness (──), and the indirect effect through physical symptoms (----) and through negative emotions (─ · ─). Latent variables are represented as circles, observed variables as squares, and pathways with arrows

All SEM models were adjusted for sex and age and are presented in Figs. 2 and 3. Model 1 investigated the direct effect of RA status on eCRF. Model 2a builds on Model 1 by adding the latent variable “physical symptoms” as a possible mediator of the relationship between RA status and eCRF. The physical symptoms latent variable was derived from three observed variables: morning stiffness, joint pain in the past 6 months, and pain in neck, back, or hips. Model 2b added a different possible mediator of the relationship between RA status and eCRF to Model 1, namely “negative emotions”. The negative emotions latent variable was derived from the observed variables depressive symptoms and perceived stress. Model 3 combined Model 2a and 2b to explore how including both physical symptoms and negative emotions as mediators of the relationship between RA status and eCRF in the same model would impact the mediating role of each latent variable. We did not adjust for smoking habits because of the complex relationship between smoking, mental health, and physical fitness. For example, smoking is a risk factor both for developing RA and reduced eCRF but might also be a mediator of the relationship between negative emotions and eCRF.

### Ethics

The Regional Committee for Medical and Health Research Ethics approved the project (#23420). The project was performed in accordance with the Helsinki declaration. All participants provided informed consent.

## Results

In total, 227 persons with RA and 300 blood donors were included the present study, see Fig. 1. In the RA group, the overall acceptance rate to participation was 61%. RA participants were less often female (67% versus 78%, p=0.03) and younger (median age 64 versus 66 years, p=0.004) compared to the RA patients who declined or did not respond to the invitation to participate. Data to calculate acceptance rate were not collected for the controls.

The age range was 21 to 83 years for the RA patients and 19 to 75 years for the controls. Participant characteristics are presented in Table 1. The persons with RA were older, more often female, more often ever smokers, had lower eCRF, more joint symptoms, and more negative emotions compared to controls. Approximately one fourth of the RA patients and one third of the controls fulfilled the aerobic PA recommendations. Although there was no statistically significant difference in the proportion who fulfilled the aerobic PA recommendations, the RA group performed PA less frequently and at lower intensity compared to the control group. Data to calculate eCRF were missing for 27% of the RA patients, largely due to missing data for resting heart rate for 51% of the patients who mailed their questionnaire. The percentage of data missing was ≤4% for all other variables. The RA patients with missing data to calculate eCRF were not significantly different from the patients with sufficient data to calculate eCRF in terms of age, gender, disease duration, physical function, negative emotions, and physical symptoms (p>0.10 for all variables).

**Table 1:** Participant characteristics^a,b^

	RA patients n=227	Controls n=300	P-value^c^
Age (years)	64 (53, 71)	46 (35, 55)	<0.0001
Female sex	153 (67)	161 (54)	0.001
Body mass index (kg/m^2^)	26 (23, 29)	26 (23, 29)	0.96
Ever smoker	150 (66)	105 (35)	<0.001
Resting heart rate (beats per minute)	68 (62, 76)	68 (63, 73)	0.62
Degree of morning stiffness (scale 0-10)	4 (1, 6)	1 (0, 2)	<0.0001
Intensity of joint pain past 6 months (scale 0-10)	4 (2, 6)	0 (0, 2)	<0.0001
Degree of pain in neck/back/hips (scale 0-10)	4 (1, 7)	2 (0, 4)	<0.0001
Physical activity frequency			0.009
Less than once per week	24 (11)	19 (6)	
Once per week	36 (16)	28 (9)	
2-3 times per week	98 (43)	130 (44)	
Almost every day	68 (30)	122 (41)	
Physical activity average intensity^d^			<0.001
Take it easy	76 (33)	58 (19)	
Heavy breathing or sweat	117 (52)	211 (70)	
Near exhaustion	7 (3)	11 (4)	
Physical activity average duration^d^			0.27
Less than 15 minutes	5 (2)	3 (1)	
15-29 minutes	33 (15)	56 (19)	
30-60 minutes	115 (51)	168 (56)	
More than 60 minutes	48 (21)	53 (18)	
Fulfilled the EULAR aerobic physical activity recommendations	63 (28)	104 (35)	0.11
Estimated cardiorespiratory fitness (mL/kg/min)			
Overall	30.9 (±10.1)	39.8 (±7.2)	<0.0001
Women	27.8 (±7.8)	36.3 (±5.8)	<0.0001
Men	37.2 (±11.5)	44.0 (±6.5)	<0.0001
Cohen’s perceived stress scale (scale 0-40)	14 (9, 18)	10 (7, 14)	<0.0001
HADS-D (scale 0-21)	3 (1, 5)	1 (0, 3)	<0.0001
RA specific information			
Age when diagnosed (years)	48 (±15)		
Disease duration (years)	11 (5, 20)		
Positive for anti-citrullinated protein antibody	167 (74)		
Positive for rheumatoid factor	159 (70)		
Patient global assessment (mm, scale 0-100)	29 (14, 48)		
mHAQ (scale 0.00-3.00)	0.25 (0.00, 0.63)		
Degree of joint tenderness or swelling (scale 0-10)	2 (1, 5)		
Medication - current use			
Conventional DMARDs	186 (82)		
Biological DMARDs	116 (51)		
Glucocorticoids	51 (22)		
Comorbidities			
Hypertension	68 (30)		
Respiratory disease^e^	43 (19)		
Cardiovascular disease^f^	42 (19)		
Cancer	22 (10)		
Diabetes	14 (6)		

^a^Presented as number with percentage, median with 25^th^ and 75^th^ percentile, or mean with standard deviation as appropriate

^b^Abbreviations: DMARDs disease-modifying antirheumatic drugs, EULAR European Alliance of Associations for Rheumatology, HADS-D Hospital Anxiety and Depression Scale’s Depression Score, mHAQ modified Health Assessment Questionnaire, RA rheumatoid arthritis

^c^Chi-square test, Mann Whitney U-test or t-test as appropriate

^d^Only participants performing physical activity at least weekly were asked to report intensity and duration

^e^Respiratory disease: chronic obstructive pulmonary disease, chronic restrictive pulmonary disease, or asthma

^f^Cardiovascular disease: myocardial infarction, angina, heart failure, arrhythmia, or stroke

Results from the SEM models are presented in Table 2 and in more detail in Online Resource Table S1-4. The unstandardized coefficient for the direct effect of RA on eCRF in Model 1 was -1.7 (95% confidence interval: -2.8 to -0.6, p=0.002). Thus, having RA was associated with statistically significantly lower eCRF and the age- and sex-adjusted mean difference in eCRF was -1.7 mL/kg/min. The indirect pathways between RA status and eCRF through physical symptoms and negative emotions were statistically significant in Model 2a and 2b, respectively. When both latent variables were included in Model 3, the standardized coefficients for both indirect pathways were -0.034 with p-values close to 0.05 (p=0.051 for physical symptoms, p=0.039 for negative emotions). Of the total effect of RA on eCRF in Model 3, 26% was the direct effect, 37% was mediated through physical symptoms, and 37% was mediated through negative emotions. In other words, 74% of the total effect of RA on eCRF was mediated by the latent variables in the final model. The fit indices for each SEM model are presented in Online Resource Table S5. The models had non-significant chi square tests, RMSEA <0.04, CFI >0.99 and TLI >0.98. The only exception was the chi-square test for Model 3, with p=0.047. As the chi-square test often is significant in large samples, model fit was considered acceptable [28].

**Table 2:** Results from the structural equation models^a^

	Associations between rheumatoid arthritis status and estimated cardiorespiratory fitness	Unstandardized coefficient (95 % confidence interval)	Standardized coefficient	P-value
Model 1	Direct effect	-1.7 (-2.8, -0.6)	-0.089	0.002
Model 2a	Direct effect	-0.9 (-2.0, 0.3)	-0.045	0.14
	Indirect effect via physical symptoms	-0.8 (-1.3, -0.3)	-0.042	0.002
	Total effect	-1.7 (-2.8, -0.6)	-0.087	0.003
Model 2b	Direct effect	-0.8 (-2.0, 0.4)	-0.041	0.20
	Indirect effect through negative emotions	-0.9 (-1.5, -0.4)	-0.048	0.002
	Total effect	-1.7 (-2.8, -0.6)	-0.089	0.002
Model 3	Direct pathway	-0.5 (-1.3, 0.0)	-0.024	0.46
	Indirect effect through physical symptoms	-0.7 (-1.3, 0.0)	-0.034	0.051
	Indirect effect through negative emotions	-0.6 (-1.3, 0.0)	-0.034	0.039
	Total effect	-1.8 (-2.8, -0.7)	-0.092	0.001

^a^Model 1: The effect of rheumatoid arthritis status on estimated cardiorespiratory fitness. Model 2a: The effect of rheumatoid arthritis status on estimated cardiorespiratory fitness, directly and indirectly through physical symptoms. Model 2b: The effect of rheumatoid arthritis status on estimated cardiorespiratory fitness, directly and indirectly through negative emotions. Model 3: The effect of rheumatoid arthritis status on estimated cardiorespiratory fitness, directly and indirectly through physical symptoms and negative emotions. All models were adjusted for age and sex

## Discussion

The main finding was that both physical symptoms and negative emotions acted as mediators of the association between RA status and eCRF in the present study. As expected, the RA patients had significantly lower eCRF compared to the controls, also when adjusting for sex and age. The indirect effect through physical symptoms and the indirect effect through negative emotions were of similar effect size. To our knowledge this is the first study to explore factors mediating the relationship between RA status and physical fitness.

### Cardiorespiratory fitness

The RA patients had comparable CRF estimates to previous CRF results in Norwegian RA patients, though higher than results in British RA patients [7, 9, 15]. The differences may be explained by recruitment strategy, recruitment year, regional and cultural factors related to PA habits, disease activity, and comorbidities. The mean difference in eCRF between RA patients and controls was smaller than expected, but in the expected direction and statistically significant. In a large Norwegian population-based cohort, the age-adjusted mean eCRF in RA patients was 3.2-5.0 mL/kg/min lower in women and 1.8-4.0 mL/kg/min lower in men compared to controls [10]. Perhaps the fitness level among Norwegian RA patients has improved over the past decade with better medical treatment. Another explanation may be participation bias in the present study. Inactive patients are less likely to participate in a study about PA especially if it involves a physical test, and perhaps the invitation to the optional 6-minute walk test for a subgroup of the RA patients made some more reluctant to participate. However, many participants with low PA levels agreed to participate as only 28% of RA patients fulfilled the aerobic PA recommendations. The eCRF results among the controls were comparable to CRF results published in healthy individuals of similar mean age in the same region of Norway [29].

The standardized coefficients for the effect of RA status on eCRF might appear small. However, the size of standardized coefficients depend on the underlying distribution and relative importance of each variable. This association is still of clinical interest as even small improvements in CRF have positive effects on long-term health [1].

As the RA participants in the present study had high self-reported function and relatively high eCRF estimates, they might not be representative for all RA patients or RA patients in other countries. However, we expect that the role of mediating variables would be even more prominent in a population with larger differences in CRF between RA patients and controls.

### Physical symptoms

Physical symptoms, represented by a latent variable based on the observed variables morning stiffness, joint pain, and pain in neck, back, or hips, explained 37% of the association between RA status and eCRF in the final model. Both pain and stiffness have been reported as important barriers to PA by RA patients [30]. Stiffness has been associated with PA habits in one study, whereas the association between pain and PA habits appears to be non-significant in most studies of RA patients [31, 32]. In patients with fibromyalgia more pain has been associated with lower fitness [33]. We may speculate that pain mainly affects exercise intensity, as our RA patients reported lower PA intensity than controls and as PA at low intensity would lead to lower CRF compared to PA at higher intensity. Regardless, exercise may lead to some reduction in pain among persons with RA. Thus, exercise can affect pain and pain can affect exercise habits [14, 30]. As the physical symptoms variable was based on three distinct symptoms, the results are not directly comparable to previous studies. The symptoms included were chosen as they are closely related to each other and therefore fit as indicators of the same latent variable. Furthermore, musculoskeletal symptoms are common both in RA patients and in healthy individuals.

Pain and morning stiffness may be prominent symptoms even when the patient is in clinical remission. EULAR recommends a patient-centred approach to pain management that involves proper assessment, a personalized management plan, and patient education [34]. Treatment of pain may involve non-pharmacological options such as PA, cognitive-behavioural therapy, and weight management, and if necessarily also pharmacological options. Some patients fear that PA can lead to disease exacerbation and joint damage [30]. This concern should be addressed with patient education, clear exercise advice, and possibly referral to a physiotherapist or other interventions that can further increase the patients’ knowledge and confidence regarding PA and exercise [16, 35].

### Negative emotions

Negative emotions, represented by a latent variable combining depressive symptoms and perceived stress, explained 37% of the association between RA status and eCRF in the final model. The relationship between RA, depression, stress, and CRF is complex. RA itself increases the risk of depression and some types of stress, however both depression and stress may increase the risk of developing RA [17, 18, 36, 37]. Both depression and stress have been associated with a reduction in PA over time, and stress has been associated with lower self-reported fitness levels [19, 38, 39]. However, high CRF protects against developing depressive symptoms [2].

Depression has several detrimental effects in RA patients, including lower chance of remission, lower adherence to treatment, higher pain levels, lower quality of life, and even increased mortality rates [17]. Stress in RA patients is associated with more pain, anxiety, and reduced quality of life [40, 41]. Active behaviour coping strategies might buffer some of the impact of perceived stress on depressive symptoms in RA patients [41]. EULAR recommends regular assessment of mental health in persons with inflammatory joint diseases and initiation of interventions if necessary, as better mental health can lead to better self-management [35]. The present study demonstrated that negative emotions are associated with lower eCRF. We speculate that improvements in mental health, particularly if the individual has high levels of depressive symptoms and perceived stress, could lead to increased PA engagement, which again may increase CRF levels. Healthy lifestyle choices such as a healthy diet, regular PA, enough sleep, strong social connections, and avoidance of risky substance intake, are beneficial for mental health [42]. Persons with clinical depression might need psychological and pharmacological interventions as well. Spending time in nature and mind-body practices are other strategies that may alleviate stress [42].

### Other mediating factors

Other factors than physical symptoms and negative emotions probably also mediate the relationship between RA and low CRF. Potential mediators might include fatigue, comorbidities, lung function, body composition, inflammation, exercise habits, anxiety, motivation, self-efficacy, coping strategies, and sarcopenia [11, 32, 43, 44]. We focused on factors that are common both in RA patients and the general population, though generally more prevalent in RA patients. Including too many potentially mediating factors will lead to a very complex model and is only feasible in very large studies. RA-specific variables such as disease duration, seropositivity, tender and swollen joint counts, or disease activity, could not be included as potential mediators due to collinearity with RA status. For example, we would not expect the controls to have swollen joints, so including swollen joint count as part of the physical symptoms latent variable would lead to collinearity with RA. Although the presence of RA in the present study was associated with lower eCRF, other factors like sex, age, PA habits, and genetics probably have a larger impact on an individual’s CRF than RA status [11, 12].

### Strengths and limitations

A strength of this study is that we included a relatively large sample of RA patients and controls recruited from the same geographical region. Even if the blood donors are a healthy sample of the population with few comorbidities and might not be directly comparable to persons with RA, both groups consisted of persons with a wide age range with varying levels of PA, and the analyses were adjusted for age and sex. The models had good fit. Nevertheless, there are likely other SEM models with acceptable fit, especially since several of the factors in the model may act bidirectionally.

Missing data for eCRF in 27% of the RA patients is a weakness. This was due to changes in the recruitment process to also include mail-only participation during the COVID-19 pandemic, which resulted in missing data for resting heart rate and consequently eCRF. However, all participants were included in the SEM models with their available data, which is a recognized strategy to deal with missing data that generally introduces less bias than for example listwise deletion [45]. Using non-exercise models to calculate eCRF can lead to both over- and underestimation of physical fitness. A cardiopulmonary exercise test would have given more accurate CRF results. Nevertheless, we did not have the resources to perform such testing. Moreover, by not including such testing less effort was required to participate, which probably resulted in a more representative and larger sample. Physical symptoms, depressive symptoms, and perceived stress are subjective variables and there is inevitably some uncertainty with these measures. HADS-D and Cohen’s perceived stress scale were chosen to assess the negative emotions because they are well-known, validated, and frequently used instruments [25, 26]. Due to the cross-sectional design, evaluation of causality was not possible.

## Conclusion

Both physical and emotional factors mediated the association between RA and low eCRF. It is important to assess or estimate CRF, or alternatively PA levels, and support persons with low eCRF in increasing their PA engagement. Both physical and psychological factors impact human behaviour, and both aspects need to be addressed for optimal care.

## Supplementary Information

Below is the link to the electronic supplementary material.

Supplementary file 1 (PDF 94.9 kb)

Supplementary file 2 (PDF 105 kb)

Supplementary file 3 (PDF 125 kb)

Supplementary file 4 (PDF 137 kb)

Supplementary file 5 (PDF 36.6 kb)

Supplementary file 6 (PDF 85.7 kb)

## Data Availability

No additional data are available.
