# Supplementary material for: The association between rheumatoid arthritis and reduced estimated cardiorespiratory fitness is mediated by physical symptoms and negative emotions: a cross-sectional study
Source: Clin Rheumatol. 2023 Mar 24;42(7):1801–10. doi: 10.1007/s10067-023-06584-x (PMC10038374; doi:10.1007/s10067-023-06584-x)
Supplement: Supplementary file 1 — (PDF 94.9 kb) [file 10067_2023_6584_MOESM1_ESM.pdf]

Online Resource Table S1

Article: The association between rheumatoid arthritis and reduced estimated cardiorespiratory fitness is mediated by physical symptoms and negative emotions: a cross-sectional study

Journal: Clinical Rheumatology.

Authors: Ingrid Sæther Houge, Mari Hoff, Vibeke Videm

Corresponding author: Professor Vibeke Videm MD PhD, Department of Clinical and Molecular Medicine, Lab Centre 3 East

St. Olavs hospital, NO-7006 Trondheim, Norway, Tel: +47 72 57 33 21, e-mail: vibeke.videm@ntnu.no

Online Resource Table S1: Detailed results from Structural Equation Model 1 [a,b]

	**Unstandardized coefficients (95% confidence interval)**			**Standardized coefficients**		
	Direct effect	Indirect effect	Total effect	Direct effect	Indirect effect	Total effect
Effect on eCRF						
Rheumatoid arthritis	-1.70 (-2.79, -0.61)*	-	-1.70 (-2.79, -0.61)*	-0.089*	-	-0.089*
Male sex	9.45 (8.51, 10.38)*	0.25 (0.05, 0.46)†	9.70 (8.76, 10.63)*	0.488*	0.013†	0.501*
Age	-0.38 (-0.42, -0.35)*	-0.03 (-0.05,-0.01)*	-0.41 (-0.44, -0,38)*	-0.645*	-0.046*	-0.691*
Effect on rheumatoid arthritis						
Male sex	-0.15 (-0.22, - 0.08)*	-	-0.15 (-0.22, - 0.08)*	-0.148*	-	-0.148*
Age	0.02 (0.01, 0.02)*	-	0.02 (0.01, 0.02)*	0.516*	-	0.516*

[a]Abbreviations: eCRF estimated cardiorespiratory fitness. †<0.05, *<0.01.

[b]Model 1: The effect of rheumatoid arthritis status on estimated cardiorespiratory fitness in a model adjusted for age and sex.
