# Supplementary material for: The association between rheumatoid arthritis and reduced estimated cardiorespiratory fitness is mediated by physical symptoms and negative emotions: a cross-sectional study
Source: Clin Rheumatol. 2023 Mar 24;42(7):1801–10. doi: 10.1007/s10067-023-06584-x (PMC10038374; doi:10.1007/s10067-023-06584-x)
Supplement: Supplementary file 2 — (PDF 105 kb) [file 10067_2023_6584_MOESM2_ESM.pdf]

Online Resource Table S2

Article: The association between rheumatoid arthritis and reduced estimated cardiorespiratory fitness is mediated by physical symptoms and negative emotions: a cross-sectional study

Journal: Clinical Rheumatology.

Authors: Ingrid Sæther Houge, Mari Hoff, Vibeke Videm

Corresponding author: Professor Vibeke Videm MD PhD

Department of Clinical and Molecular Medicine, Lab Centre 3 East

St. Olavs hospital, NO-7006 Trondheim, Norway

Tel: +47 72 57 33 21, e-mail: vibeke.videm@ntnu.no

Online Resource Table S2: Detailed results from Structural Equation Model 2a [a,b]

	Unstandardized coefficients (95% confidence interval)			Standardized coefficients		
	Direct effect	Indirect effect	Total effect	Direct effect	Indirect effect	Total effect
Effect on eCRF						
Rheumatoid arthritis	-0.87 (-2.03, 0.30)	-0.81 (-1.33, -0.29)*	-1.68 (-2.77, -0.59)*	-0.045	-0.042*	-0.087*
Male sex	9.26 (8.34, 10.19)*	0.25 (0.05, 0.45)†	9.51 (8.59, 10.43)*	0.480*	0.013†	0.493*
Age	-0.38 (-0.41, -0.35)*	-0.03 (-0.05, -0.01)*	-0.41 (-0.44, -0.38)*	-0.640*	-0.055*	-0.695*
Physical symptoms	1 (constrained)	-	1 (constrained)	0.095*	-	0.095*
Effect on rheumatoid arthritis						
Male sex	-0.15 (-0.22, -0.08)*	-	-0.15 (-0.22, -0.08)*	-0.148*	-	-0.148*
Age	0.02 (0.01, 0.02)*	-	0.02 (0.01, 0.02)*	0.516*	-	0.516*
Effect on physical symptoms						
Rheumatoid arthritis	-0.81 (-1.33, -0.29)*	-	-0.81 (-1.33, -0.29)*	-0.444*	-	-0.444*
Male sex	-	0.12 (0.02, 0.22)†	0.12 (0.02, 0.22)†	-	0.066†	0.066†
Age	-0.01 (-0.01, 0.00)	-0.01 (-0.02, 0.00)*	-0.02 (-0.03, -0.01)*	-0.099	-0.229*	-0.328*
Effect on joint pain past six months						
Rheumatoid arthritis	-	2.42 (1.94, 2.91)*	2.42 (1.94, 2.91)*	-	0.423*	0.423*
Physical symptoms	-2.99 (-4.83, -1.15)*	-	-2.99 (-4.83, -1.15)*	-0.952*	-	-0.952*
Sex	-	-0.36 (-0.55, -0.17)*	-0.36 (-0.55, -0.17)*	-	-0.062*	-0.062*
Age	-	0.06 (0.04, 0.07)*	0.06 (0.04, 0.07)*	-	0.313*	0.313*
Effect on morning stiffness						
Rheumatoid arthritis	-	2.26 (1.81, 2.71)*	2.26 (1.81, 2.71)*	-	0.399*	0.399*
Physical symptoms	-2.79 (-4.51, -1.08)*	-	-2.79 (-4.51, -1.08)*	-0.899*	-	-0.899*
Male sex	-	-0.34 (-0.51, -0.16)*	-0.34 (-0.51, -0.16)*	-	-0.060*	-0.060*
Age	-	0.05 (0.04, 0.06)*	0.05 (0.04, 0.06)*	-	0.295*	0.295*
Effect on pain in neck/back/hips						
Rheumatoid arthritis	-	1.71 (1.22, 2.19)*	1.71 (1.22, 2.19)*	-	0.287*	0.287*
Physical symptoms	-2.11 (-3.33, -0.89)*	-	-2.11 (-3.33, -0.89)*	-0.647*	-	-0.647*
Male sex	-	-0.25 (-0.40, -0.11)*	-0.25 (-0.40, -0.11)*	-	-0.042*	-0.042*
Age	-	0.04 (0.03, 0.05)*	0.04 (0.03, 0.05)*	-	0.212*	0.212*

[a]Abbreviations: eCRF estimated cardiorespiratory fitness. †<0.05. *<0.01.

[b]Model 2a: The effect of rheumatoid arthritis status on estimated cardiorespiratory fitness, directly and indirectly through physical symptoms, in a model adjusted for age and sex.
