# Supplementary material for: The association between rheumatoid arthritis and reduced estimated cardiorespiratory fitness is mediated by physical symptoms and negative emotions: a cross-sectional study
Source: Clin Rheumatol. 2023 Mar 24;42(7):1801–10. doi: 10.1007/s10067-023-06584-x (PMC10038374; doi:10.1007/s10067-023-06584-x)
Supplement: Supplementary file 3 — (PDF 125 kb) [file 10067_2023_6584_MOESM3_ESM.pdf]

Online Resource Table S3

Article: The association between rheumatoid arthritis and reduced estimated cardiorespiratory fitness is mediated by physical symptoms and negative emotions: a cross-sectional study

Journal: Clinical Rheumatology.

Authors: Ingrid Sæther Houge, Mari Hoff, Vibeke Videm

Corresponding author: Professor Vibeke Videm MD PhD

Department of Clinical and Molecular Medicine, Lab Centre 3 East

St. Olavs hospital, NO-7006 Trondheim, Norway

Tel: +47 72 57 33 21, e-mail: vibeke.videm@ntnu.no

Online Resource Table S3: Detailed results from Structural Equation Model 2b [a,b]

	**Unstandardized coefficients (95% confidence interval)**			**Standardized coefficients**		
	Direct effect	Indirect effect	Total effect	Direct effect	Indirect effect	Total effect
Effect on eCRF						
- Rheumatoid arthritis	-0.78 (-1.99, 0.42)	-0.93 (-1.50, -0.35)*	-1.71 (-2.81 -0.62)*	-0.041	-0.048*	-0.089*
- Male sex	9.50 (8.56, 10.44)*	0.25 (0.05, 0.46)†	9.75 (8.82, 10.69)*	0.490*	0.013†	0.503*
- Age	-0.39 (-0.43, -0.36)*	-0.02 (-0.04, 0.00)	-0.41 (-0.44, -0.38)*	-0.661*	-0.029	-0.690*
- Negative emotions	1 (constrained)	-	1 (constrained)	0.107*	-	0.107*
Effect on rheumatoid arthritis						
- Male sex	-0.15 (-0.22, -0.08)*	-	-0.15 (-0.22, -0.08)*	-0.148*	-	-0.148*
- Age	0.02 (0.01, 0.02)*	-	0.02 (0.01, 0.02)*	0.516*	-	0.516*
Effect on negative emotions						
- Rheumatoid arthritis	-0.93 (-1.50, -0.35)*	-	-0.93 (-1.50, -0.35)*	-0.453*	-	-0.453*
- Male sex	-	0.14 (0.03, 0.25)†	0.14 (0.03, 0.25)†	-	0.067†	0.067†
- Age	0.01 (0.00, 0.02)†	-0.02 (-0.02, -0.01)*	-0.01 (-0.01, 0.00)	0.157†	-0.234*	-0.077
Effect on HADS-D						
- Rheumatoid arthritis	-	2.35 (1.80, 2.89)*	2.35 (1.80, 2.89)*	-	0.406*	0.406*
- Negative emotions	-2.52 (-4.00, 1.05)*	-	-2.52 (-4.00, -1.05)*	-0.897*	-	-0.897*
- Sex	0.23 (-0.24, 0.71)	-0.35 (-0.54, -0.16)*	-0.12 (-0.61, 0.38)	0.040	-0.060*	-0.020
- Age	-	0.01 (0.00, 0.03)	0.01 (0.00, 0.03)	-	0.069	0.069
Effect on perceived stress scale						
- Rheumatoid arthritis	-	4.18 (2.99, 5.37)*	4.18 (2.99, 5.37)*	-	0.334*	0.334*
- Negative emotions	-4.50 (-7.03, -1.96)*	-	-4.50 (-7.03, -1.96)*	-0.737*	-	-0.737*
- Male sex	-1.30 (-2.34, -0.26)†	-0.62 (-0.98, -0.27)*	-1.92 (2.99, -0.85)*	-0.103†	-0.049*	-0.152*
- Age	-	0.02 (0.00, 0.05)	0.02 (0.00, 0.05)	-	0.057	0.057

[a]Abbreviations: eCRF estimated cardiorespiratory fitness, HADS-D Hospital Anxiety and Depression Scale' Depression Score. †<0.05. *<0.01.

[b]Model 2b: The effect of rheumatoid arthritis status on estimated cardiorespiratory fitness, directly and indirectly through negative emotions, in a model adjusted for age and sex.
