# Supplementary material for: The association between rheumatoid arthritis and reduced estimated cardiorespiratory fitness is mediated by physical symptoms and negative emotions: a cross-sectional study
Source: Clin Rheumatol. 2023 Mar 24;42(7):1801–10. doi: 10.1007/s10067-023-06584-x (PMC10038374; doi:10.1007/s10067-023-06584-x)
Supplement: Supplementary file 4 — (PDF 137 kb) [file 10067_2023_6584_MOESM4_ESM.pdf]

Online Resource Table S4

Article: The association between rheumatoid arthritis and reduced estimated cardiorespiratory fitness is mediated by physical symptoms and negative emotions: a cross-sectional study

Journal: Clinical Rheumatology.

Authors: Ingrid Sæther Houge, Mari Hoff, Vibeke Videm

Corresponding author: Professor Vibeke Videm MD PhD

Department of Clinical and Molecular Medicine, Lab Centre 3 East

St. Olavs hospital, NO-7006 Trondheim, Norway

Tel: +47 72 57 33 21, e-mail: vibeke.videm@ntnu.no

Online Resource Table S4: Detailed results from Structural Equation Model 3 [a,b]

	Unstandardized coefficients (95% confidence interval)			Standardized coefficients		
	Direct effect	Indirect effect	Total effect	Direct effect	Indirect effect	Total effect
Effect on eCRF						
- Rheumatoid arthritis	-0.46 (-1.70, 0.77)	-1.30 (-1.98, -0.62)*	-1.76 (-2.85, -0.68)*	-0.024	-0.068*	-0.092*
- Male sex	9.30 (8.37, 10.22)*	0.26 (0.06, 0.47)†	9.56 (8.64, 10.48)*	0.480*	0.014†	0.494*
- Age	-0.39 (-0.42, -0.35)*	-0.02 (-0.05, 0.00)†	-0.41 (-0.44, -0.38)*	-0.652*	-0.043†	0.695*
- Physical symptoms	1 (constrained)	-	1 (constrained)	0.072†	-	0.072†
- Negative emotions	-0.27 (-0.53, -0.02)†	-	-0.27 (-0.53, -0.02)†	-0.074†	-	-0.074†
Effect on rheumatoid arthritis						
- Male sex	-0.15 (-0.22, -0.08)*	-	-0.15 (-0.22, -0.08)*	-0.148*	-	-0.148*
- Age	0.02 (0.01, 0.02)*	-	0.02 (0.01, 0.02)*	0.516*	-	0.516*
Effect on physical symptoms						
- Rheumatoid arthritis	-0.65 (-1.31, 0.00)	-	-0.65 (-1.31, 0.00)	-0.471	-	-0.471
- Male sex	-	0.10 (-0.01, 0.21)	0.10 (-0.01, 0.21)	-	0.070	0.070
- Age	-0.01 (-0.01, 0.00)	-0.01 (-0.02, 0.00)	-0.02 (-0.03, 0.00)	-0.101	-0.243	-0.344
Effect on joint pain past six months						
- Rheumatoid arthritis	-	2.38 (1.90, 2.86)*	2.38 (1.90, 2.86)*	-	0.416*	0.416*
- Physical symptoms	-3.65 (-7.28, -0.03)†	-	-3.65 (-7.28, -0.03)†	-0.882†	-	-0882†
- Sex	-	-0.36 (-0.54, -0.17)*	-0.36 (-0.54, -0.17)*	-	-0.061*	-0.061*
- Age	-	0.05 (0.04, 0.07)*	0.05 (0.04, 0.07)*	-	0.304*	0.304*
Effect on morning stiffness						
- Rheumatoid arthritis	-	2.25 (1.80, 2.71)*	2.25 (1.80, 2.71)*	-	0.398*	0.398*
- Physical symptoms	-3.46 (-6.89, -0.03)†	-	-3.46 (-6.89, -0.03)†	-0.845†	-	-0.845†
- Sex	-	-0.34 (-0.51, -0.16)*	-0.34 (-0.51, -0.16)*	-	-0.059*	-0.059*
- Age	-	0.05 (0.04, 0.06)*	0.05 (0.04, 0.06)*	-	0.291*	0.291*
Effect on pain in neck/back/hips						
- Rheumatoid arthritis	-	1.94 (1.49, 2.40)*	1.94 (1.49, 2.40)*	-	0.327*	0.327*
- Physical symptoms	-2.98 (-5.90, -0.07)†	-	-2.98 (-5.90, -0.07)†	-0.694†	-	-0.694†
- Sex	-	-0.29 (-0.45, -0.13)*	-0.29 (-0.45, -0.13)*	-	-0.048*	-0.048*
- Age	-	0.04 (0.03, 0.06)*	0.04 (0.03, 0.06)*	-	0.239*	0.239*
Effect on negative emotions						
- Rheumatoid arthritis	2.36 (1.82, 2.91)*	-	2.36 (1.82, 2.91)*	0.456*	-	0.456*
- Male sex	-	-0.35 (-0.54, -0.16)*	-0.35 (-0.54, -0.16)*	-	-0.067*	-0.067*

- Age	-0.03 (-0.04, -0.01)*	0.04 (0.03, 0.05)*	0.01 (0.00, 0.03)	-0.159*	0.236*	0.077
Effect on HADS-D						
- Rheumatoid arthritis	-	2.36 (1.82, 2.91)*	2.36 (1.82, 2.91)*	-	0.409*	0.409*
- Negative emotions	1 (constrained)	-	1 (constrained)	0.897*	-	0.897*
- Sex	0.34 (-0.11, 0.79)	-0.35 (-0.54, -0.16)*	-0.01 (-0.48, 0.46)	0.058	-0.060*	-0.002
- Age	-	0.01 (0.00, 0.03)	0.01 (0.00, 0.03)	-	0.069	0.069
Effect on perceived stress scale						
- Rheumatoid arthritis	-	4.23 (3.02, 5.43)*	4.23 (3.02, 5.43)*	-	0.338*	0.338*
- Negative emotions	1.79 (1.39, 2.18)*	-	1.79 (1.39, 2.18)*	0.740*	-	0.740*
- Male sex	-1.11 (-2.10, -0.11)†	-0.63 (-0.99, -0.27)*	-1.73 (-2.77, -0.70)*	-0.088†	-0.050*	-0.138*
- Age	-	0.02 (0.0, 0.05)	0.02 (0.00, 0.05)	-	0.057	0.057

[a]Abbreviations: eCRF estimated cardiorespiratory fitness, HADS-D Hospital Anxiety and Depression Scale' Depression Score. †<0.05. *<0.01.

[b]Model 3: The effect of rheumatoid arthritis status on estimated cardiorespiratory fitness, directly and indirectly through physical symptoms and negative emotions, in a model adjusted for age and sex
