# Supplementary material for: The association between rheumatoid arthritis and reduced estimated cardiorespiratory fitness is mediated by physical symptoms and negative emotions: a cross-sectional study
Source: Clin Rheumatol. 2023 Mar 24;42(7):1801–10. doi: 10.1007/s10067-023-06584-x (PMC10038374; doi:10.1007/s10067-023-06584-x)
Supplement: Supplementary file 5 — (PDF 36.6 kb) [file 10067_2023_6584_MOESM5_ESM.pdf]

Online Resource Table S5

Article: The association between rheumatoid arthritis and reduced estimated cardiorespiratory fitness is mediated by physical symptoms and negative emotions: a cross-sectional study

Journal: Clinical Rheumatology.

Authors: Ingrid Sæther Houge, Mari Hoff, Vibeke Videm

Corresponding author: Professor Vibeke Videm MD PhD

Department of Clinical and Molecular Medicine, Lab Centre 3 East

St. Olavs hospital, NO-7006 Trondheim, Norway

Tel: +47 72 57 33 21, e-mail: vibeke.videm@ntnu.no

Online Resource Table S5: Model fit indices for the structural equation models [a]

	P-value for the chi-square test	Root mean square error of approximation	Comparative fit index	Tucker Lewis Index
Model 1	Not applicable	<0.001	1.000	1.000
Model 2a	0.15	0.032	0.998	0.993
Model 2b	0.25	0.026	0.999	0.995
Model 3	0.047	0.037	0.995	0.987

[a]Model 1: The effect of rheumatoid arthritis status on estimated cardiorespiratory fitness. Model 2a: The effect of rheumatoid arthritis status on estimated cardiorespiratory fitness, directly and indirectly through physical symptoms. Model 2b: The effect of rheumatoid arthritis status on estimated cardiorespiratory fitness, directly and indirectly through negative emotions. Model 3: The effect of rheumatoid arthritis status on estimated cardiorespiratory fitness, directly and indirectly through physical symptoms and negative emotions. All models were adjusted for age and sex.
