# Supplementary material for: The association between rheumatoid arthritis and reduced estimated cardiorespiratory fitness is mediated by physical symptoms and negative emotions: a cross-sectional study
Source: Clin Rheumatol. 2023 Mar 24;42(7):1801–10. doi: 10.1007/s10067-023-06584-x (PMC10038374; doi:10.1007/s10067-023-06584-x)
Supplement: Supplementary file 6 — (PDF 85.7 kb) [file 10067_2023_6584_MOESM6_ESM.pdf]

Online Resource Text 1

Article: The association between rheumatoid arthritis and reduced estimated cardiorespiratory fitness is mediated by physical symptoms and negative emotions: a cross-sectional study

Journal: Clinical Rheumatology.

Authors: Ingrid Sæther Houge, Mari Hoff, Vibeke Videm

Corresponding author: Professor Vibeke Videm MD PhD

Department of Clinical and Molecular Medicine, Lab Centre 3 East

St. Olavs hospital, NO-7006 Trondheim, Norway

Tel: +47 72 57 33 21, e-mail: vibeke.videm@ntnu.no

Model fit indices and estimation method

Common model fit indices in structural equation modelling (SEM) are the chi-square test, root mean error of approximation (RMSEA), comparative fit index (CFI), and Tucker-Lewis index (TLI) [1]. The chi square test compares the SEM model to a model that fits the data perfectly. RMSEA is related to the residual between the model and the observed data, accounting for sample size and model complexity. CFI and TLI estimates how much better the SEM model fits the data compared to the baseline model that assumes no correlation between the variables, penalizing for complex models in different ways. A model with that fit the data well should have a non-significant chi-square test, RMSEA <0.10, CFI ≥0.90, and TLI ≥0.90 [1].

The estimation method maximum likelihood with missing values was chosen in the present study as many participants missed data for one or more variables. This method includes all participants in the analysis with each participant contributing with their available data.

References

1) Mehmetoglu M, Jakobsen T. Applied statistics using Stata. A guide for the social sciences: Sage Publications 2017:270-322.
